# Gliomas and Farm Pesticide Exposure in Women: The Upper Midwest Health Study

**DOI:** 10.1289/ehp.7456

**Published:** 2005-02-09

**Authors:** Tania Carreón, Mary Ann Butler, Avima M. Ruder, Martha A. Waters, Karen E. Davis-King, Geoffrey M. Calvert, Paul A. Schulte, Barbara Connally, Elizabeth M. Ward, Wayne T. Sanderson, Ellen F. Heineman, Jack S. Mandel, Roscoe F. Morton, Douglas J. Reding, Kenneth D. Rosenman, Glenn Talaska, the Brain Cancer Collaborative Study Group

**Affiliations:** ^1^National Institute for Occupational Safety and Health, Cincinnati, Ohio, USA;; ^2^National Cancer Institute, National Institutes of Health, Department of Health and Human Services, Rockville, Maryland, USA;; ^3^School of Public Health, University of Minnesota, Minneapolis, Minnesota, USA;; ^4^Mercy Foundation, Des Moines, Iowa, USA;; ^5^Natural Farm Medicine Center, Marshfield Clinic, Marshfield, Wisconsin, USA;; ^6^Department of Medicine, Michigan State University, East Lansing, Michigan, USA;; ^7^Department of Environmental Health, University of Cincinnati, Cincinnati, Ohio, USA

**Keywords:** brain cancer, case—control, farmers, glioma, Midwest, pesticides, women

## Abstract

An excess incidence of brain cancer in male farmers has been noted in several studies, but few studies have focused on women. The National Institute for Occupational Safety and Health Upper Midwest Health Study evaluated effects of rural exposures for 341 female glioma cases and 528 controls, all adult (18–80 years of age) nonmetropolitan residents of Iowa, Michigan, Minnesota, and Wisconsin. On average, controls lived longer on farms than did cases. After adjusting for age, age group, education, and farm residence, no association with glioma was observed for exposure to arsenicals, benzoic acids, carbamates, chloroacetanilides, dinitroanilines, inorganics, organochlorines, organophosphates, phenoxys, triazines, or urea-based or estrogenic pesticides. An increased risk of glioma was observed for carbamate herbicides but was not statistically significant (odds ratio = 3.0; 95% confidence interval, 0.9–9.5). No association was observed between glioma and exposure to 12 widely used specific pesticides, after adjustment for age, age group, education, and any other pesticide exposure. These results were not affected after exclusion of proxy respondents (43% of cases, 2% of controls). Women were less likely than men to have applied pesticides, but more likely to have laundered pesticide-contaminated clothes. Storing pesticides in the house was associated with a statistically non-significant increased risk. Results show that exposure to pesticides was not associated with an increased risk of intracranial gliomas in women. Other farm-related factors could be etiologic factors and will be discussed in future reports.

Several studies have shown that working on a farm or in the agricultural industry may lead to higher risks of brain cancer in men (Brownson et al. 1990; Morrison et al. 1992; Reif et al. 1989; Rodvall et al. 1996; Zheng et al. 2001). Research on specific agricultural factors associated with higher risks of brain cancer has led to several studies that have documented pesticide exposures as etiologic factors in men (Bohnen and Kurland 1995; Musicco et al. 1988).

The National Institute for Occupational Safety and Health (NIOSH) developed the Upper Midwest Health Study (UMHS), a case–control study of intracranial gliomas among rural residents. The primary hypothesis of the UMHS was that pesticides would be associated with increased risk of brain cancer. This study focused on one type of brain tumor (gliomas), which is the most common type in adults (Inskip et al. 1995). Gliomas were studied to increase the homogeneity of the case group, in contrast with previous studies that have combined different types of brain tumors with likely different etiologies. Furthermore, brain gliomas appear to be more related to occupational risk factors than are other types of brain cancer (Schlehofer et al. 1990).

Among men, we found that exposure to farm insecticides, fumigants, and organochlorine pesticides was associated with reduced glioma risk [insecticides: adjusted odds ratio (OR) = 0.5; 95% confidence interval (CI), 0.4–0.8; fumigants: OR = 0.6; 95% CI, 0.4–0.9; organochlorines: OR = 0.7; 95% CI, 0.5–0.9]. Modest increased risks were associated with exposure to carbamate fungicides and herbicides and to dinitroanilines.

Few studies have evaluated the relationship between pesticide exposure and brain cancer in women. Cocco et al. (1999) reported an increased risk of mortality from cancer of the brain and other parts of the central nervous system, including gliomas, in women reportedly exposed to insecticides and fungicides (OR = 1.3; 95% CI, 1.1–1.5). Zheng et al. (2001) reported a significant increase in the risk of glioma among women employed in farm occupations in Iowa (OR = 4.1; 95% CI, 1.0–17.9). Neither of these studies, however, evaluated exposure to pesticides grouped by chemistry (e.g., carbamates), only by function (e.g., insecticides). This article focuses on the epidemiologic evidence for the association of pesticide exposures and gliomas in women.

## Materials and Methods

The study population included women 18–80 years of age residing in four states (Iowa, Michigan, Minnesota, and Wisconsin) in counties where the largest population center had < 250,000 residents. Cases with a histologically confirmed primary intracranial glioma [*International Classification of Diseases for Oncology* (ICD-O) codes 938–948] (Percy et al. 1990) diagnosed from 1 January 1995 through 31 January 1997, were identified through participating medical facilities and neurosurgeon offices by a rapid ascertainment system to try to complete case eligibility determination and physician consent in 2–3 weeks. Cases with a prior malignancy other than a glioma were not excluded. Physician consent was obtained before contacting cases or their next of kin. Case ascertainment completeness was determined by comparing with the corresponding glioma cases in state cancer registries in all four states. Ascertainment percentages were 78.2% for Iowa, 82.7% for Michigan, 86.5% for Minnesota, and 65.5% for Wisconsin.

Controls were women with no diagnosis of glioma. Controls with a prior diagnosis of cancer or any other disease were not excluded. They were randomly selected within 10-year age-group strata, with the proportion/stratum determined by the age distribution of glioma cases in that state from 1992 through 1994. The target was to recruit 1.5 controls per case. Controls were frequency matched within the state but not by county of residence; however, only those counties from which cases were selected were considered for the selection of controls.

Controls between 18 and 64 years of age were selected from the state driver’s license/non-driver identification records (unpublished data) and those between 65 and 80 years of age from Health Care Financing Administration’s (now Centers for Medicare & Medicaid Services) Medicare data (unpublished data). Selection of controls was based on the expected number of cases and their distribution by age and sex from the tumor registry data available in each state during a 3-year period (1989–1992). An algorithm was developed for control selection. Once potential controls were identified, their addresses were verified and telephone numbers obtained.

After mailing requests for participation, interviewers telephoned to arrange interview appointments. Refusants were asked to complete a brief telephone interview of critical questions. Before the interview, the interviewer administered informed consent.

Enclosed with a letter confirming the interview appointment were two lists of pesticides for the respondent to review before the interview. These lists were based on research on crops grown and pesticides used in recent years in the participating study states (Sanderson et al. 1997). Participants who ever lived or worked on farms were asked to report their lifetime agricultural pesticide exposures until 1 January 1993. This date was the cutoff for all histories and other exposure information, because more recent exposures would not be relevant to etiology. The particular date chosen was near the beginning of a new presidential term, a life event all respondents likely would remember. Exposure to specific pesticides was queried first (ever exposed), prompted by the pesticide lists. We collected data on years of pesticide use, application days, or acreage covered only for those applying pesticides directly. Questions covering a wide range of farm activities, including washing pesticide-contaminated clothes and whether specific crops were grown or animals were raised were asked only of those who lived or worked on a farm after age 18. The questionnaire, modified for use in the present study, was based on one developed by the National Cancer Institute (Chen et al. 2002).

An industrial hygienist reviewed occupational histories in the completed questionnaire and selected follow back questionnaires that were sent to the respondents, and answers obtained by telephone. To minimize recall bias, we used a standardized interview, used the same methods of interviewing cases and controls, provided intensive interviewer training, and kept the interviewers blind to the study hypotheses. Periodic brief telephone reinterviews, focusing on critical questions, were compared with full questionnaires, and interviewers were retrained as necessary to eliminate discrepancies.

Proxy respondents (individuals who provide information on behalf of someone else) were used when the study participant had died or was too impaired to answer the questionnaire. Because of variability in the quality of the information provided by surrogates, we endeavored to use proxy respondents who had knowledge of the subjects’ exposure history. In our analyses, we did not make a statistical adjustment for the type of respondent (although we recorded who answered the questionnaire). We did, however, perform separate analyses and calculate ORs using data only from participants and from participants and proxies combined.

All estimates of association were adjusted through stratification or multivariate modeling. Because 97% of study subjects and state residents were white, race was not used as a matching variable. Age-adjusted ORs and 95% CIs first were computed to determine whether the hypothesized risk factors were associated with an increased odds for developing intracranial brain gliomas and to help determine potential confounders to the associations of interest. Age adjustment included a lineal term. ORs and 95% CIs were estimated by unconditional logistic regression models. All the analyses used SAS software version 8.0 (SAS Institute Inc., Cary, NC).

All participants and other respondents will be notified of the overall study results. This study was approved by the NIOSH Human Subjects Review Board and by review boards at every participating institution, and was conducted in accordance with subsection (m) of the Privacy Act of 1974 (5 U.S.C. 552a) and Section 308(d) of the Public Health Service Act (42 U.S.C. 242m) to safeguard individuals and establishments against invasions of privacy.

## Results

The potential study population included 1,257 women: 476 cases and 781 controls. Case and control review eliminated 98 ineligible cases and 45 ineligible controls. The final study pool included 378 eligible cases and 736 eligible controls, among whom 341 cases (90.2%) and 527 controls (71.6%) participated in the study. Among the participants, 196 (57%) of cases and 516 (98%) of controls were direct respondents; for the others, proxy respondents were used. Most proxy respondents were spouses (35%), child or children (31%), parent(s) (4%), or sibling(s) (3%). Twenty percent of proxy interviews involved two or more of these close relatives, and only 1% did not include a first-degree relative. The mean time between diagnosis and interview for cases was 26 weeks (range, 3–162 weeks).

Of the 341 cases, 56.6% were glioblastomas (ICD-O codes 9440–9441), 21.7% were astrocytomas (ICD-O 9400–9424), 12.9% were oligodendrogliomas (ICD-O 9450–9451), 5.9% were mixed gliomas or malignant gliomas not otherwise specified (ICD-O 9380–9381), and the rest (2.9%) had other histologic outcomes.

We performed correlation analyses comparing control distribution by county with the population distribution by county in 1992. High correlations were observed (> 0.85, *p* < 0.0001) for all the controls and when they were stratified by state. Similar correlations were observed (> 0.79, *p* < 0.0001) when the analyses included only those 18–80 years of age and when stratified by sex. Based on these observations, we can conclude that controls were similar to the general population in age and sex distribution by county and state.

Table 1 shows demographic characteristics and cigarette smoking history of study participants. Controls were, on average, older than cases (*p* < 0.0005). For this reason, all analyses are shown adjusted by age. Also, because controls were frequency matched by age to the cases, all the models included the age strata as indicator variables. Models were also adjusted for schooling, as an indicator of socioeconomic status. Other characteristics, such as race, allergies, and number of pregnancies, did not differ significantly between cases and controls. Adjustment for these factors did not have an effect on the risk estimates.

More than half of the cases and the controls reported ever living or working on a farm (Table 2). This finding was expected given the selection procedure. Cases reported an average of 22.6 (± 17.0) total farm years and controls spent an average of 25.1 (± 19.8) years, and this difference was statistically significant (*p* = 0.03).

Among those who ever lived on a farm, approximately 60% indicated that pesticides were used on those farms. No association was observed between the use of any pesticide and glioma (OR = 1.1; 95% CI, 0.8–1.6). Use of insecticides, herbicides, or fungicides was not associated with an increased risk of glioma, nor were other farm practices reported in Table 2. Participants handling pesticides on nonfarming jobs were not at a higher risk of glioma (OR = 1.2; 95% CI, 0.6–2.2), but women who held nonfarming jobs for a year or more had a marginally increased risk (OR = 1.5; 95% CI, 0.8–2.8).

Compared with men from the same study, women were less likely to apply pesticides (8% in women vs. 29% in men). Conversely, more women than men reported laundering pesticide-contaminated clothes (20% in women vs. 3% in men). Neither of these practices was associated with an increase in the risk of glioma (Table 2). Storing pesticides in the house increased the relative risk of glioma, but the association was not statistically significant.

Reported pesticide trade names were associated with the appropriate generic name(s) using a relational database. The NIOSH pesticide reference database (Ruder AM, unpublished data), expanded from the pesticide lists sent to participants before the interviews, was used to associate trade name responses with the appropriate generic name(s). For example, Bronco, Bullet, Cannon, Freedom, Lariat, Lasso, and Saddle would all link to alachlor. Pesticides were classified into 13 broad categories, based on their chemical properties. A total of 82 female participants (33 cases and 49 controls) reported exposure to pesticides not included in any of the categories. Table 3 shows adjusted ORs associated with exposure to pesticide categories, including and excluding proxy responses. The baseline comparison is to those cases and controls who reported having not used any pesticide. Exposures to pesticides in the 12 categories were not associated with an increased risk of glioma. Results were not affected by exclusion of proxy responses. A 3-fold risk of glioma was observed among women exposed to carbamate herbicides (Table 3), but the association did not reach statistical significance, likely as a result of the small number of people exposed to this pesticide category.

Table 4 presents the ORs for glioma due to exposure to the 12 individual pesticides most commonly used by the whole study population (men and women). The comparison group for each pesticide includes those cases and controls who reported not being exposed to any pesticide. No increased risk of glioma was associated with exposure to any particular pesticide. Similar results were observed for pesticides with reported estrogenic activities [alachlor and dichlorodiphenyltrichloroethane (DDT); Klotz et al. 1996]. These results did not change after excluding proxy responses.

## Discussion

In this analysis, focusing only on women, no association was found between exposure to pesticides grouped in different categories and the risk of intracranial glioma. Likewise, exposure to individual pesticides did not increase the risk of glioma. Farm-related factors, such as farm residence, size of farm, contact with animals, use of solvents, washing of pesticide-applicator clothes, and storage of pesticides at home, did not have an effect on the risk of glioma. Only women exposed to carbamate herbicides appeared to have an increased risk of glioma, but the number of people exposed was low.

This is, to our knowledge, the first study to evaluate the effect of farm pesticide exposure on intracranial glioma in women. A major strength of this study is the large number of cases (*n* = 341) and controls (*n* = 527). This study also incorporated an extensive questionnaire on farm and rural risk factors, and pesticide use and exposure. A thorough process of identification of generic pesticides allowed classification of pesticides into major categories.

Another strength of the study is the systematic selection of cases, limited to histologically confirmed newly diagnosed primary gliomas. Also, exclusion of other brain malignancies, such as meningiomas, eliminated the variability associated with different types of brain cancer.

A limitation of our study was that controls were, on average, older than cases. Because we used the distribution of glioma cases by age and sex in the 3 years preceding the study period (because the distribution within our study period was not available until it ended) to select controls, case–control differences in age distribution were possible. The effect of age could influence our analyses and provide a biased estimate of risk. We corrected for this difference in all analyses by adjusting for age and age group stratum. Results did not differ from those obtained in crude analyses, indicating that the older age of controls did not likely have an effect on the risk estimates.

Another limitation of this study is the use of proxy respondents, a common concern in studies of brain cancer, given the incapacitating nature of the disease. In this study, proxy respondents accounted for 43% of the cases, but only 2% of the controls, introducing the possibility of differential misclassification bias between cases and controls. The percentage of proxy interviews in this study is lower than the 74% obtained by Zheng et al. (2001), comparable with the 46% obtained by Lee et al. (1997), and higher than the 16% obtained by Inskip et al. (2001) in studies of risk factors for brain tumors. The study by Inskip et al. (2001) was hospital based and therefore more likely to recruit cases before they die or become too ill to respond directly.

In a study designed to compare information on agricultural factors obtained by interview from farmers and their proxy respondents, Blair et al. (1995) reported that the reliability of proxy reports for general issues such as educational level, years living on a farm, or years engaged in farming, was relatively high. However, reliability of proxy responses was poor for questions on details of agricultural practices. Other researchers have reached similar conclusions (Coggon et al. 1985; Gardner et al. 2002), suggesting that risk estimates would tend to shift toward the null. In a study that evaluated proxy reliability in a case–control study of rural and agricultural risk factors and Parkinson disease, spouses and adult children were fairly reliable informants concerning these exposures (Wang et al. 1994). We tried to account for the effect of proxy interviews by comparing the results with and without proxy responses, and did not observe differences. Our findings suggest that the use of proxies did not have an effect on the risk estimates, but results should be interpreted with caution because they may be affected by selection bias.

In this study, the retrospective assessment of exposure relied on the participants’ or their proxies’ memory. With the passage of time, it is more difficult for respondents to recall details of exposure, including specific pesticide use and frequency of use. It has been suggested, however, that farmers’ personal involvement in the many tasks involved in pest control may reinforce memory, improving recall at some later date (Blair and Zahm 1990). Because no objective measurements of pesticide exposure were available, exposure undoubtedly would be misclassified for some subjects. Since we do not have an indication that differential recall between cases and controls occurred in this study, an underestimation of associations is possible, presenting a problem in our study because we found no increased risk.

Our study does not support the hypothesis that farm residence or pesticide exposure increases the risk of glioma. Other studies have suggested increased brain cancer risks among female farmers. Heineman et al. (1995) observed an increased risk of brain tumors among women employed as grain farmers in Shanghai, China. That study, however, did not report information on specific pesticides or other agricultural exposures and was not limited to gliomas. Cocco et al. (1999) reported a higher risk of mortality from central nervous system cancer due to exposure to pesticides; however, no trend was observed with increased exposure to these chemicals. Most recently, Zheng et al. (2001) reported an increased risk of glioma in women involved in farming and other agricultural occupations in Iowa, one of the states included in the present study. A stronger association was reported for those women who reported working in these occupations for more than 10 years. The study, however, did not evaluate exposure to pesticides. A meta-analysis of eight studies in farmers who were females or female relatives of farmers who assisted in farming found no association with brain cancer (type not specified) (OR = 1.0; 95% CI, 0.8–1.3) (Khuder et al. 1998), showing agreement with the results of our study. It is possible, however, that the effect is different for different types of brain tumors, and therefore our results could not be comparable.

The International Agency for Research on Cancer (IARC) has concluded that arsenic and arsenical compounds (including pesticides) are carcinogenic to humans (IARC 1987). In this study, arsenical pesticides did not increase the risk of glioma. However, to our knowledge, there are no studies that have shown an association between arsenical pesticides and glioma. IARC has also concluded that spraying and application of nonarsenical insecticides “entail exposures that are probably carcinogenic to humans” (IARC 1987). Furthermore, Hu et al. (1999) have provided evidence that arsenic exposures from pesticides are far lower than those from indoor occupational exposures (e.g., smelter workers).

Conversely, IARC has not evaluated the carcinogenicity of butylate and *S*-ethyl dipropylthiocarbamate, the carbamate herbicides most commonly used by study participants. In this study, we found some evidence of an increased risk of glioma in women exposed to carbamate herbicides. Additional work would be needed to elucidate any carcinogenicity.

Pesticides have different chemical structures and therefore different toxicity modes of action. A number of factors affect pesticide absorption and metabolism and ultimately may affect their toxicity, such as anatomical site, chemical properties, and environmental factors (temperature, humidity, and occlusion) (Hodgson 2001). These factors could not be considered in our statistical analyses. Moreover, simultaneous exposure to two or more chemicals may have an effect on the induction or inhibition of enzymes involved in the metabolism of pesticides, and ultimately in their carcinogenicity (Hodgson 2001; Hodgson and Levi 2001). Therefore, studies that take into account factors that affect the metabolism of pesticides are warranted. A potential explanation of the lack of consistency between this study and previously published studies is that increased risk may not be due exclusively to pesticide exposure, but to interactions among a variety of agricultural exposures and additional factors that alter susceptibility (McDuffie 1994).

It has been suggested that hormonal factors play a role in the development of brain tumors, particularly meningiomas in women (not considered here) (Huang et al. 2004). Some pesticides have been reported to be endocrine disruptors, that is, interfere with hormone function (Solomon and Schettler 2000). In this study, we did not find evidence that pesticides with reported estrogenic activities, such as DDT and alachlor, increase the risk of gliomas in women. This finding is consistent with the sparse presence of estrogen receptors in intracranial gliomas tissue samples (Paoletti et al. 1990).

Finally, it is important to recognize that women need to be studied separately from men when agricultural factors are involved. Most studies on the effect of agricultural practices on health have been conducted in men; in women, the association has been explored in only a few studies (Cocco et al. 1998; Heineman et al. 1995; Ronco et al. 1992; Schlehofer et al. 1990; Zheng et al. 2001). There is a commonly held perception that women typically are not involved in farming and agricultural activities (McDuffie 1994). Numerous studies, however, have documented the wide variety of farming tasks done by women, even women defining themselves as homemakers (McCoy et al. 2002; Reed et al. 1999). As shown in this study, women were less likely than men to apply pesticides themselves. However, farm women were exposed to pesticides by living on their farms, engaging sporadically in agricultural practices, and laundering pesticide-applicator clothes. It has been shown that laundering does not remove pesticide residue and therefore constitutes an important source of exposure (Laughlin et al. 1984).

Furthermore, even though many of the health effects of pesticides are the same for men and women, sex-related biologic differences strongly support a distinct susceptibility to their toxic action (Garcia 2003). Possible causes for this difference include hormone-related processes, patterns of storage of lipophilic pesticides due to the higher levels of adipose tissue in women, and women’s life events such as pregnancy, lactation, or menopause.

In summary, results from this study show that exposure to farm pesticides was not associated with an increased risk of intracranial gliomas in women. Because effect underestimation cannot be ruled out as a possible explanation, these results warrant further investigation. Other farm-related factors, such as exposure to solvents or fertilizers, could be etiologic factors and will be discussed in future reports from this study.

## Figures and Tables

**Table 1:** Characteristics of female participants in the UMHS, cases and controls [no. (%)].

	Including proxy respondents		Excluding proxy respondents	
Characteristic	Cases (*n* = 341)	Controls (*n* = 527)	Cases (*n* = 196)	Controls (*n* = 516)
Age*^a^* (years)				
15–30	43 (13)	54 (10)	37 (19)	54 (10)
31–40	58 (17)	50 (9)	49 (25)	50 (10)
41–50	52 (15)	63 (12)	33 (17)	63 (12)
51–60	60 (18)	97 (18)	30 (15)	96 (19)
61–70	85 (25)	179 (34)	35 (18)	175 (34)
71–80	43 (13)	84 (16)	12 (6)	78 (15)
State of residence				
Iowa	76 (22)	143 (27)	47 (24)	136 (27)
Michigan	114 (33)	133 (25)	62 (32)	130 (25)
Minnesota	67 (20)	116 (22)	41 (21)	115 (22)
Wisconsin	84 (25)	135 (26)	46 (23)	135 (26)
Ethnicity: white non-Latina	335 (98)	519 (98)	193 (98)	509 (99)
Education				
College graduate	52 (15)	74 (14)	35 (18)	74 (14)
High school graduate	236 (69)	375 (71)	143 (73)	368 (71)
< 12 years	53 (16)	78 (15)	18 (9)	74 (14)
Smoking history				
Never smoked	180 (53)	307 (58)	107 (55)	300 (58)
Ex-smoker	83 (32)	130 (30)	39 (27)	128 (30)
Current (1993) smoker	78 (23)	90 (17)	50 (25)	88 (17)
Ever drank alcohol	212 (62)	321 (61)	129 (66)	317 (61)
Ever pregnant	300 (88)	458 (87)	169 (86)	449 (87)
No. of pregnancies				
1–2	100 (33)	134 (29)	58 (34)	134 (30)
3–4	120 (40)	177 (39)	68 (40)	171 (38)
5–7	64 (21)	118 (26)	37 (22)	116 (26)
≥8	16 (5)	29 (6)	6 (4)	28 (6)
Menstruating through 1992	130 (38)	159 (30)	112 (57)	159 (31)

a Age in 1993. Eligibility requirement was age > 18 years at time of diagnosis or control selection. The ranges include women diagnosed with glioma after 1993 and therefore in 1993 still possibly < 18 years of age. Controls had to be 18 by 1 January 1995 so they also could have been < 18 years of age in 1993.

**Table 2:** Farm-related practices and risk of glioma among women, cases and controls [no. (%)].

	Including proxy respondents			Excluding proxy respondents		
Characteristic	Cases	Controls	OR*^a^* (95% CI)	Cases	Controls	OR*^a^* (95% CI)
Ever lived/worked on farm	186 (54)	313 (59)	1.0 (0.7–1.3)	98 (50)	306 (59)	0.9 (0.6–1.3)
Years on farm						
≤10	38 (21)	74 (24)	Referent	26 (27)	73 (24)	Referent
11–20	76 (41)	100 (32)	1.7 (1.0–2.8)	38 (39)	99 (32)	1.4 (0.7–2.6)
21–30	26 (14)	45 (14)	1.3 (0.7–2.5)	12 (12)	45 (15)	1.1 (0.5–2.6)
31–40	15 (8)	24 (8)	1.3 (0.6–2.8)	7 (7)	23 (7)	1.1 (0.4–3.2)
41–50	11 (6)	20 (6)	1.3 (0.5–3.1)	6 (6)	20 (6)	1.3 (0.4–3.8)
> 50	18 (10)	50 (16)	0.9 (0.4–1.8)	8 (8)	46 (15)	1.0 (0.4–2.7)
Farm acreage						
≤40	27 (17)	33 (12)	1.3 (0.6–2.9)	14 (16)	32 (12)	1.0 (0.4–2.8)
41–80	26 (17)	44 (16)	1.4 (0.6–3.4)	14 (16)	44 (17)	1.0 (0.4–3.0)
81–160	46 (29)	83 (31)	1.3 (0.6–3.0)	27 (31)	82 (31)	1.2 (0.5–3.1)
161–240	23 (15)	43 (16)	1.3 (0.6–2.8)	11 (13)	41 (16)	1.3 (0.6–3.1)
241–360	18 (11)	30 (11)	1.8 (0.8–4.1)	9 (10)	30 (11)	1.4 (0.5–3.7)
> 361	16 (10)	33 (12)	Referent	11 (13)	31 (12)	Referent
Herbicides ever used on farm	70 (38)	114 (38)	1.0 (0.6–1.5)	41 (42)	112 (39)	1.0 (0.6–1.7)
Insecticides ever used on farm	100 (55)	155 (52)	1.2 (0.8–1.8)	57 (59)	152 (53)	1.6 (0.9–2.7)
Fungicides ever used on farm	14 (8)	20 (7)	1.2 (0.6–2.4)	7 (7)	20 (7)	0.9 (0.4–2.5)
Fumigants ever used on farm	11 (6)	37 (12)	0.4 (0.2–0.9)	7 (7)	37 (13)	0.6 (0.2–1.4)
Living on farm as adult (≥ 18 years of age)	117 (64)	189 (64)	1.1 (0.7–1.6)	58 (60)	184 (64)	1.0 (0.6–1.7)
Cattle, hogs, or chickens raised*^b^*	103 (88)	176 (93)	0.6 (0.3–1.4)	52 (90)	172 (94)	1.0 (0.3–3.0)
Solvents used to clean hands*^b^*	14 (12)	23 (12)	1.0 (0.4–2.0)	11 (19)	23 (12)	1.4 (0.6–3.3)
Laundered pesticide-applicator clothes*^b^*	57 (69)	114 (76)	0.7 (0.4–1.3)	29 (63)	113 (77)	0.5 (0.2–1.2)
Pesticides stored in house*^b^*	6 (8)	6 (5)	2.0 (0.6–6.8)	4 (10)	6 (5)	2.7 (0.7–11.0)

a Adjusted for age, 10-year age group, education, farm residence.

b Question asked only of subjects living on farm after 18 years of age.

**Table 3:** Exposure to pesticide categories and risk of glioma in women, cases and controls (no.).

	Including proxy respondents			Excluding proxy respondents		
Pesticide category	Cases	Controls	OR*^a^* (95% CI)	Cases	Controls	OR*^a^* (95% CI)
No pesticide exposure (farm, home, or job)	156	200	—	96	197	—
Arsenicals	13	27	1.0 (0.5–1.9)	8	26	1.5 (0.7–3.7)
Benzoic acids	13	29	0.8 (0.4–1.5)	8	28	0.9 (0.4–2.1)
Carbamates	15	29	1.0 (0.5–1.9)	10	28	1.2 (0.6–2.8)
Fungicides	4	5	1.6 (0.4–6.5)	2	5	1.3 (0.2–7.5)
Herbicides	8	5	3.0 (0.9–9.5)	5	5	3.5 (0.9–13.0)
Insecticides	11	25	0.8 (0.4–1.8)	8	24	1.3 (0.4–2.0)
Chloroacetanilides	21	33	1.1 (0.6–2.0)	12	33	1.0 (0.4–2.0)
Dinitroanilines	14	31	0.8 (0.4–1.5)	9	30	0.8 (0.4–1.9)
Inorganics	9	16	0.8 (0.3–2.1)	5	16	0.7 (0.2–2.2)
Organochlorines	42	70	1.2 (0.7–1.8)	20	70	1.1 (0.6–2.0)
Organophosphates	35	67	0.9 (0.6–1.5)	23	67	1.1 (0.6–2.0)
Herbicides	18	41	0.7 (0.4–1.3)	10	41	0.6 (0.3–1.2)
Insecticides	29	55	0.9 (0.6–1.6)	21	55	1.4 (0.8–2.6)
Phenoxys	25	51	0.9 (0.5–1.5)	12	50	0.7 (0.4–1.5)
Triazines	32	56	1.0 (0.6–1.7)	19	54	1.1 (0.6–2.1)
Urea-based	3	7	0.6 (0.2–2.6)	2	7	0.7 (0.1–3.6)
Estrogenic	52	76	1.4 (0.9–2.2)	27	76	1.4 (0.8–2.5)

a Adjusted for age, 10-year age group, education, and any other pesticide exposure.

**Table 4:** Exposure to individual pesticides and risk of glioma in women, cases and controls (no.).

	Including proxy respondents			Excluding proxy respondents		
Pesticide CAS number	Cases	Controls	OR*^a^* (95% CI)	Cases	Controls	OR*^a^* (95% CI)
No pesticide exposure	156	200	—	96	197	—
2,4-D, CAS 94-75-7	24	46	0.9 (0.5–1.6)	11	45	0.8 (0.4–1.6)
Alachlor,*^b^* CAS 15972-60-8	20	28	1.2 (0.7–2.3)	11	28	1.0 (0.5–2.3)
Atrazine, CAS 1912-24-9	32	52	1.1 (0.7–1.8)	19	50	1.2 (0.6–2.3)
Bentazon, CAS 25057-89-0	9	14	1.1 (0.4–2.6)	7	14	1.4 (0.5–3.8)
Cyanazine, CAS 21725-46-2	11	24	0.7 (0.3–1.6)	9	23	1.1 (0.5–2.5)
DDT,*^c^* CAS 50-29-3	36	55	1.3 (0.8–2.2)	18	55	1.5 (0.8–2.8)
Diazinon, CAS 333-41-5	18	26	1.3 (0.7–2.5)	13	26	1.9 (0.9–4.1)
Dicamba, CAS 1918-00-9	11	26	0.7 (0.3–1.5)	8	25	1.0 (0.4–2.5)
Glyphosate, CAS 1071-83-6	18	41	0.7 (0.4–1.3)	10	41	0.6 (0.3–1.2)
Imazethapyr, CAS 81335-77-5	8	16	0.8 (0.3–1.9)	6	16	0.9 (0.3–2.4)
Malathion, CAS 121-75-5	18	35	1.0 (0.5–1.8)	13	35	1.5 (0.7–3.0)
Metolachlor, CAS 51218-45-2	9	17	0.8 (0.3–1.9)	5	17	0.7 (0.2–2.1)
Pendimethalin, CAS 40487-41-1	8	9	1.4 (0.5–3.9)	6	9	1.7 (0.5–5.2)
Trifluralin, CAS 1582-09-8	10	23	0.7 (0.3–1.6)	5	22	0.6 (0.2–1.6)

Abbreviations: 2,4-D, 2,4-dichlorodiphenoxyacetic acid; CAS, Chemical Abstracts Service.

a Adjusted for age, 10-year age group, education, and any other pesticide exposure.

b Weak estrogenic activity.

c Estrogenic activity.
